# Information-geometric measures estimate neural interactions during oscillatory brain states

**DOI:** 10.3389/fncir.2014.00011

**Published:** 2014-02-24

**Authors:** Yimin Nie, Jean-Marc Fellous, Masami Tatsuno

**Affiliations:** ^1^Department of Neuroscience, Canadian Centre for Behavioural Neuroscience, University of LethbridgeLethbridge, AB, Canada; ^2^Department of Psychology, Program in Applied Mathematics, University of ArizonaTucson, AZ, USA

**Keywords:** information geometry, spikes, spiking neuron model, oscillation, neural networks

## Abstract

The characterization of functional network structures among multiple neurons is essential to understanding neural information processing. Information geometry (IG), a theory developed for investigating a space of probability distributions has recently been applied to spike-train analysis and has provided robust estimations of neural interactions. Although neural firing in the equilibrium state is often assumed in these studies, in reality, neural activity is non-stationary. The brain exhibits various oscillations depending on cognitive demands or when an animal is asleep. Therefore, the investigation of the IG measures during oscillatory network states is important for testing how the IG method can be applied to real neural data. Using model networks of binary neurons or more realistic spiking neurons, we studied how the single- and pairwise-IG measures were influenced by oscillatory neural activity. Two general oscillatory mechanisms, externally driven oscillations and internally induced oscillations, were considered. In both mechanisms, we found that the single-IG measure was linearly related to the magnitude of the external input, and that the pairwise-IG measure was linearly related to the sum of connection strengths between two neurons. We also observed that the pairwise-IG measure was not dependent on the oscillation frequency. These results are consistent with the previous findings that were obtained under the equilibrium conditions. Therefore, we demonstrate that the IG method provides useful insights into neural interactions under the oscillatory condition that can often be observed in the real brain.

## Introduction

The dynamics of neural interactions have been conjectured to play an important role in neural information processing. One way to investigate the neural interactions is to record multi-neuronal firing activity from a freely behaving animal, and analyze the correlations between individual units. In past decades, electrophysiological studies have significantly been advanced by the use of multi-electrode recording techniques (Wilson and McNaughton, 1993; Chapin et al., 1999; Kudrimoti et al., 1999; Laubach et al., 2000; Hoffman and McNaughton, 2002; Buzsaki, 2004; Tatsuno et al., 2006; Euston et al., 2007; Davidson et al., 2009; Peyrache et al., 2009b; Dragoi and Tonegawa, 2011, 2013). In order to analyze such high-dimensional multi-neuronal datasets, a number of statistical methods have also been developed (Gerstein and Perkel, 1969; Abeles and Gerstein, 1988; Aertsen et al., 1989; Zhang et al., 1998; Panzeri and Schultz, 2001; Grun et al., 2002a,b; Brown et al., 2004; Fellous et al., 2004; Czanner et al., 2005; Shimazaki and Shinomoto, 2007; Amari, 2009; Gilestro et al., 2009; Peyrache et al., 2009a; Shimokawa and Shinomoto, 2009; Lopes-Dos-Santos et al., 2011). Recently, a method based on information geometry (IG) has been applied to the analysis of neural data (Amari and Nagaoka, 2000; Amari, 2001; Nakahara and Amari, 2002; Amari et al., 2003; Tatsuno and Okada, 2004; Eleuteri et al., 2005; Ikeda, 2005; Miura et al., 2006; Nakahara et al., 2006; Gilestro et al., 2009; Tatsuno et al., 2009; Ince et al., 2010; Lovette et al., 2011; Nie and Tatsuno, 2012). It has been demonstrated that IG provides a powerful statistical tool for analyzing spiking data. Some of the advantages of IG approach include the orthogonal decomposition of neural interactions (Amari, 2001; Nakahara and Amari, 2002), and its direct relationship to underlying connections (Tatsuno and Okada, 2004; Tatsuno et al., 2009; Nie and Tatsuno, 2012); the single-IG measure is related to the amount of external inputs and the pairwise-IG measure is related to the amount of direct neural interactions between two neurons.

These IG properties were often investigated under the assumption that the network is in an equilibrium state. However, in the brain, the equilibrium assumption does not hold true. Instead, the brain undergoes a variety of non-equilibrium states such as oscillations. For example, the slow-wave oscillation (~1 Hz) was discovered during non-REM sleep (Steriade et al., 1993; Crunelli and Hughes, 2010), and evidence suggests that it plays an important role in memory consolidation (Huber et al., 2004; Stickgold, 2005; Diekelmann and Born, 2010). The theta (6–10 Hz) rhythm is a prominent coherent oscillation observed in the hippocampus, and its surrounding area during rat spatial navigation (Vanderwolf, 1969; Bland, 1986; Buzsaki, 2002). The theta rhythm has also been observed in various human neocortical areas during the delay period of working memory tasks (Raghavachari et al., 2001; Meltzer et al., 2008). The beta (15–30 Hz) oscillation is conjectured to play a key role in action preparation and inhibitory control in the motor system (Baker et al., 1997). The gamma (30–80 Hz) oscillation has been shown to play a role in the integration of sensory information (Gray et al., 1989; Singer and Gray, 1995). The fast hippocampal sharp wave ripples (100–200 Hz) were also reported during an animal's awake immobility and slow-wave sleep (Buzsaki et al., 1992). Therefore, it is important to investigate if the IG measures can be applied to neural data under oscillatory conditions.

In this study, we investigated how the single- and pairwise-IG measures are influenced by a network oscillation. Under an equilibrium assumption, previous studies have shown that the single- and pairwise-IG measures provide a robust estimation of the magnitude of external input and direct neural interactions (Tatsuno et al., 2009; Nie and Tatsuno, 2012). We also focused on these IG measures in this study because the external inputs and intrinsic neural interactions are the two main factors for characterizing network dynamics. For the oscillation mechanisms, we have considered two representative cases; one is an external driven oscillation where a network is influenced by external oscillatory inputs. The other is an internally induced oscillation where interactions between excitatory and inhibitory neuron populations produce an oscillation. By computer simulations using simple binary model neurons or more biologically plausible spiking neurons, we investigated whether the properties of the IG measures that were established with the equilibrium condition still hold true under oscillatory network states.

In section Methods, we briefly introduce an information-geometric analysis of neural spikes. In section Results, we describe the model and network structure used in the numerical simulation. In section Discussion, the simulation results for both externally driven and internally induced oscillations are described in detail. In section Acknowledgments, we summarize our findings and discuss future directions of research on this topic.

## Methods

### Information-geometric method

We briefly introduce the information-geometric method for spiking data analysis (for details see Amari and Nagaoka, 2000). Generally, in an *N*-neuron system, the state of *i*-th(*i* = 1, …, *N*) neuron is represented by a binary random variable *x*_*i*_, where *x*_*i*_ = 1 or 0 representing neuronal firing or silence, respectively. The joint probability distribution of the *N*-neuron system can be described by a fully expanded *N*-th order log-linear model (LLM)

(1) $$\begin{array}{l} \log\;p_{x_{1}x_{2}\cdots x_{N}}=\sum_{i}\theta_{i}^{\left(N,\;N\right)}x_{i}+\sum_{i<j}\theta_{ij}^{\left(N,\;N\right)}x_{i}x_{j}+\cdots \\ \;+\;\theta_{12,\cdots N}^{\left(N,\;N\right)}x_{1}x_{2}\cdots x_{N}-\psi {\left(\theta \right)}^{\left(N,\;N\right)}, \end{array}$$

where θ^(*N, N*)^_*ij*,… *m*_ (1 ≤ *m* ≤ *N*) represents the *m*-neuron interaction and ψ(θ)^(*N, N*)^ with θ = {θ^(*N, N*)^_*i*_, θ^(*N, N*)^_*ij*_, …, θ^(*N, N*)^_12,…*N*_ } is a normalization constant such that ∑ *p*_*x*1*x*2_ … *x*_*N*_ = 1. The first and the second superscripts in θ^(*N, N*)^_*ij*, … *m*_ represent the order of the LLM and the number of neurons in the system. We use θ^(*N, N*)^_*i*_, θ^(*N, N*)^_*ij*_, and θ^(*N, N*)^_*ij*, … *m*_ to describe the single-IG measure, the pairwise-IG measure and the *m*-neuron IG measure with the *N*-th order LLM for a *N*-neuron system, respectively, (Nie and Tatsuno, 2012). The joint probability of *N* neurons is calculated by

(2) $$p_{x_{1}x_{2},\ldots ,x_{N}}=\frac{c_{x_{1}x_{2},\ldots ,x_{\text{N}}}}{\sum_{x_{1}x_{2},\ldots ,x_{\text{N}}}c_{x_{1}x_{2},\ldots ,x_{\text{N}}}},$$

where *c*_*x*1_*x*_2_,…,*x*_*N*_ is the count of events (*X*_1_ = *x*_1_, *X*_2_ = *x*_2_, …,*X*_*N*_ = *x*_*N*_) that occur.

However, in reality, it is difficult to calculate the statistical information from all neurons in a large network. Therefore, the partially expanded LLM is often used for the estimation of neuronal interactions. The partially expanded *k*-th order LLM in an *N*-neuron network is given by

(3) $$\begin{array}{l} \log p_{x_{1}x_{2}\cdots x_{k,\;*\;\cdots \;*}}=\sum_{i}\theta_{i}^{\left(k,\;N\right)}x_{i}+\sum_{i<j}\theta_{ij}^{\left(k,\;N\right)}x_{i}x_{j}+\cdots \\ \;+\;\theta_{12,\cdots k}^{\left(k,\;N\right)}x_{1}x_{2}\cdots x_{k}-\psi {\left(\theta \right)}^{\left(k,\;N\right)} \end{array}$$

where **θ** = {θ^(*k, N*)^_*i*_, θ^(*k, N*)^_*ij*_, …, θ^(*k, N*)^_12,… *k*_}. The first few terms of **θ** and normalization factor are given as follows:

(4) $$\begin{array}{l} \theta_{i}^{\left(k,\;N\right)}=\log\frac{p_{x_{1}\;=\;0,\;\cdots ,\;x_{i}\;=\;1,\;\cdots ,\;x_{k}\;=\;0,*\cdots *}}{p_{x_{1}\;=\;0,\cdots ,x_{k}\;=\;0,*\;\cdots \;*}},\\ \theta_{ij}^{\left(k,\;N\right)}=\log\frac{\begin{array}{c} p_{x_{1}\;=\;0,\ldots ,\;x_{i}\;=\;1,\ldots ,\;x_{j}\;=\;1,\cdots ,x_{k}\;=\;0,*\;\cdots \;*}\\ p_{x_{1}\;=\;0,\cdots ,\;x_{k}\;=\;0,*\cdots *} \end{array}}{\begin{array}{c} p_{x_{1}\;=\;0,\ldots ,\;x_{i}\;=\;1,\ldots ,\;x_{j}\;=\;0,\ldots ,\;x_{k}\;=\;0,\;*\;\cdots \;*}\\ p_{x_{1}\;=\;0,\ldots ,\;x_{i}\;=\;0,\ldots ,\;x_{j}\;=\;1,\ldots ,\;x_{k}\;=\;0,\;*\cdots *\cdots \cdots \cdots } \end{array}}\\ \psi^{\left(k,\;N\right)}=-\log p_{x_{1}\;=\;0,\ldots ,\;x_{k}\;=\;0,\;*\;\cdots \;*}, \end{array}$$

where ‘* … *′, represents the marginalization over the (*N* − *k*) neurons.

The single-IG measure θ^(*k, N*)^_*i*_ and the pairwise-IG measure θ^(*k, N*)^_*ij*_ are the two main focuses in this study because θ^(*k, N*)^_*i*_ is related to the amount of external inputs and θ^(*k, N*)^_*ij*_ is related to the amount of direct neural interactions between two neurons (Tatsuno and Okada, 2004; Tatsuno et al., 2009; Nie and Tatsuno, 2012). Using a network of simple binary neurons, and the assumption of an equilibrium state, the previous study has shown that the single-IG measure θ^(2, *N*)^_*i*_ and the pairwise-IG measure θ^(2, *N*)^_*ij*_ with the 2nd-order LLM are related to the network parameters as

(5) $$\theta_{i}^{\left(2,\;N\right)}\propto 2h_{i}+O\left(\frac{1}{N}\right),\;\theta_{ij}^{\left(2,\;N\right)}\propto (J_{ij}+J_{ji})+O\left(\frac{1}{N}\right),$$

where *h*_*i*_ represents the magnitude of constant external input to a neuron *i*, and *J*_*ij*_ (*J*_*ji*_) is the connection weight from a neuron *j* to *i* (from a neuron *i* to *j*), respectively, (Tatsuno et al., 2009). If a network receives correlated inputs, the relationship for θ^(2, *N*)^_*ij*_ in Equation 5 does not hold true anymore. However, we have also shown that θ^(*k, N*)^_*ij*_ with the higher k-th order LLM provides a better estimation of neural interactions (Nie and Tatsuno, 2012). For example, θ^(4, *N*)^_*ij*_ was shown to have the relationship

(6) $$\theta_{ij}^{\left(4,\;N\right)}\propto \left(J_{ij}+J_{ji}\right),$$

within approximately a 10% error if the number of neurons *N* is ~10^3^ or larger; a typical size of network in a cortical column (Urban et al., 2001). We have also confirmed that the relationship θ^(4, *N*)^_*i*_ α2*h*_*i*_ holds true within approximately a 10% error (unpublished data).

These properties could be useful for the field of neuroscience because the IG measures can estimate the changes of underlying network parameters (*h*_*i*_ and *J*_*ij*_) separately, while other correlation measures have not yet been shown to have such a property (Amari, 2009). However, these results were derived under the equilibrium limit, and little is known if the similar relationship holds under the oscillatory condition.

### Neuron model and network structure

#### Neuron model

We investigated the influence of oscillations using a network of simple binary neurons with stochastic dynamics (Ginzburg and Sompolinsky, 1994) and biologically plausible spiking neurons (Izhikevich, 2003). Using simple binary model neurons, we first investigated whether the property of the IG measures that were shown under the equilibrium condition also held true for the oscillatory condition. We then extended our investigation to more realistic spiking neurons.

For a binary model neuron, the transition between the binary states is given by the transition rate *w* as

(7) $$\begin{array}{c} w(x_{i}=0\to x_{i}=1)=\frac{g(u_{i})}{\tau_{0}},\\ w(x_{i}=1\to x_{i}=0)=\frac{1-g(u_{i})}{\tau_{0}},\\ w(x_{i}=0\to x_{i}=0)=1-w(0\to 1),\\ w(x_{i}=1\to x_{i}=1)=1-w(1\to 0), \end{array}$$

where τ_0_ is a microscopic characteristic time and *u*_*i*_ represents the total input to the *i*-th neuron.

(8) $$g(u_{i})=\frac{1+\tanh(u_{i}-m)}{2}$$

is the sigmoidal function in the bounded interval [0, 1] where *m* is a parameter controlling the firing probability of a model neuron.

For a biologically more plausible neuron model, we adopted the Izhikevich model because it is known to be computationally efficient and biologically plausible (Izhikevich, 2003). The Izhikevich model reduced the complex dynamics of the Hodgkin–Huxley (HH) neuronal models to two coupled differential equations as

(9) $$\text{​​​}\frac{dV_{i}}{dt}=0.04V_{i}^{2}+5V_{i}+140-U_{i}+I_{i},\frac{dU_{i}}{dt}=a_{i}(b_{i}V_{i}-U_{i}).$$

Here the variable *V*_*i*_ represents the membrane potential of neuron *i*, and *U*_*i*_ represents a membrane recovery variable which correlates with the activation of *K*^+^ ionic currents and inactivation of *Na*^+^ [for detail see (Izhikevich, 2003)]. *U*_*i*_ and *V*_*i*_ are reset after a spike: if *V*_*i*_ ≥ 30 mV, then *V_*i*_* ← *c*_*i*_, *U_i_* ← *U*_*i*_ + *d*_*i*_. *I*_*i*_ represents a total input to neuron *i*; *a*_*i*_, *b*_*i*_, *c*_*i*_, *d*_*i*_ are dimensionless adjustable parameters which are usually taken as (*a*_*i*_, *b*_*i*_) = (0.02, 0.2) and (*c*_*i*_, *d*_*i*_) = (−65, 8) + (15, −6)*r*^2^_*i*_ for excitatory neurons, (*a*_*i*_, *b*_*i*_) = (0.02, 0.25) + (0.08, −0.05*r*_*i*_) and (*c*_*i*_, *d*_*i*_) = (−65, 2) for inhibitory neurons. *r*_*i*_ is a uniformly distributed random variable on the interval [0, 1] (Izhikevich, 2003).

#### Network structure

**Figure 1:**
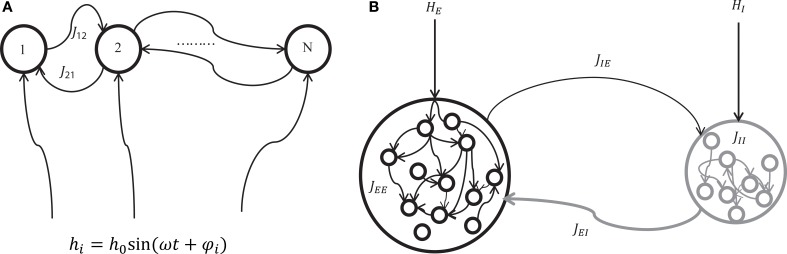
**A schematic of two mechanisms for generating network oscillations. (A)** Oscillation is generated by an external oscillatory input (externally driven oscillation). A neuron *i* in the network of *N* neurons with recurrent connections *J*_*ij*_ receives a sinusoidal external input *h*_*i*_ = *h*_0_ sin(ω*t* + φ_*i*_) where *h*_0_, ω, and φ_*i*_ represent the amplitude, angular speed, and phase of the sinusoidal input, respectively. **(B)** Oscillation is generated by the interaction between excitatory and inhibitory neuron pools (internally induced oscillation). Excitatory neurons are connected by positive connections *J*_*EE*_, inhibitory neurons are connected by negative connections *J*_*II*_, inhibitory neurons receive positive connections *J*_*IE*_ from excitatory neurons, and excitatory neurons receive negative connections *J*_*EI*_ from inhibitory neurons. In addition, excitatory and inhibitory neurons receive external constant input *H*^*E*^_*i*_ and *H*^*I*^_*k*_, respectively.

We considered two mechanisms for generating oscillatory network states; one is the oscillation driven by external inputs (Figure 1A), and the other is the oscillation induced by the intrinsic interaction between excitatory and inhibitory neuron populations (Figure 1B). The former mechanism can be a model for hippocampal theta oscillation in which the projections from the medial septum to the hippocampus play a central role (Dragoi et al., 1999). The latter structure where excitatory and inhibitory neuron pools interact is widely observed in cortical areas (Buzsaki and Wang, 2012). It can be a model for cortical oscillations (such as in a gamma-range) that rely on the interplay between excitatory and inhibitory neuron pools.

In the first scenario (externally driven oscillation, Figure 1A), a sinusoidal external input *h*_*i*_(*t*) = *h*_0_ sin(ω*t* + φ_*i*_) for the *i*-th neuron was used to generate oscillatory states in a network, where *h*_0_, ω, and *upvarphi*_*i*_ represent the amplitude, angular speed, and phase of sinusoidal signals, respectively. Note that *h*_0_ and ω are common to all neurons, but φ_*i*_ can be different for individual neurons. The explicit expression of an input signal allows one to produce different network oscillations systematically. For the binary neuron model, the total input to the *i*-th neuron is written as,

(10) $$u_{i}(t)=\sum_{j}J_{ij}x_{j}(t)+h_{i}(t).$$

where *J*_*ij*_ represents a connection weight from the *j*-th neuron to the *i*-th neuron. The neuronal state *x*_*i*_(*t*) was then updated following the transition rate *w* in Equation 7. Note that model neurons are identical, whether they are excitatory or inhibitory.

For the Izhikevich model in the first scenario, we considered a population of excitatory neurons. Although it has been demonstrated that a network of excitatory neurons can synchronize, a network of Izhikevich neurons that were connected in this particular way cannot produce an intrinsic oscillation (Mirollo and Strogatz, 1990; Hansel et al., 1995). This allows us to investigate the relationship between the IG measures and an externally driven oscillation in a more realistic setting. The total input to an Izhikevich neuron *i* is given by,

(11) $$I_{i}^{E}(t)=\sum_{j}J_{ij}^{EE}s_{j}^{E}(t)+h_{i}(t),$$

where *J*^*EE*^ represent positive weights between excitatory neurons and *s*^*E*^_*j*_ = δ(*t* − *t*^*f*^_*j*_) is the delta function representing the existence of a spike emitted from an excitatory neuron *j* at time *t*^*f*^_*j*_. The neuronal state was then updated by Equation 9 and the associated reset dynamics. In the numerical simulation, we used a small bin width of 1 ms so that it would contain no more than one spike.

**Figure 2:**
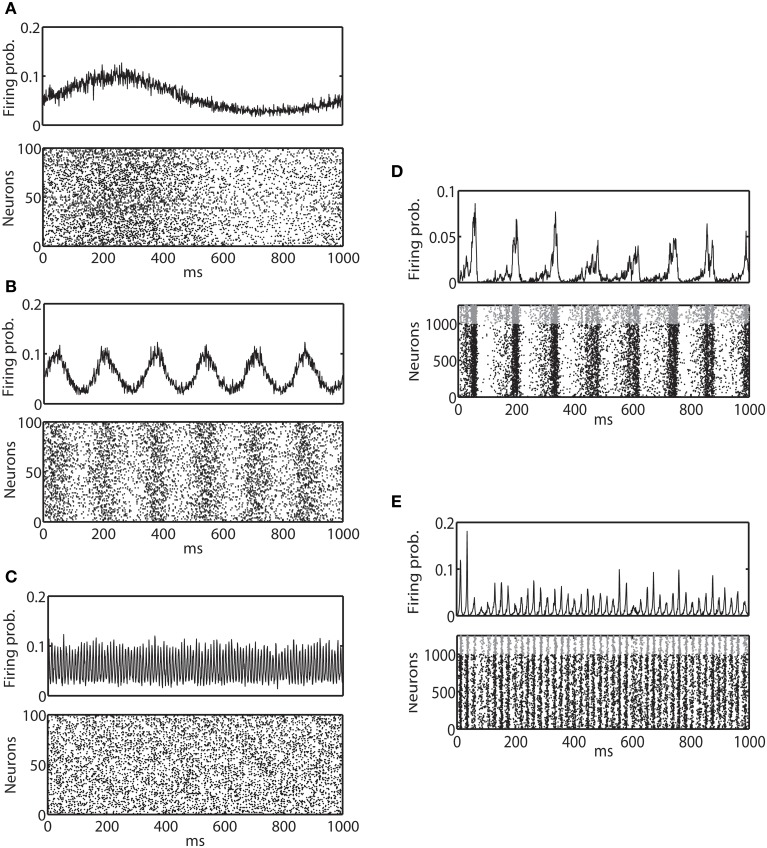
**Average firing probability and raster plot of representative oscillatory activity. (A)** Average firing probability of 1000 binary neurons (top panel) and a raster plot of 100 randomly sampled neurons (bottom panel) over 1000 ms under the influence of an external sinusoidal input of 1 Hz (slow oscillation) are shown. **(B)** Average firing probability of 1000 binary neurons (top panel) and a raster plot of 100 randomly sampled neurons (bottom panel) for an external sinusoidal input of 6 Hz (theta oscillation) are shown. **(C)** Average firing probability of 1000 binary neurons (top panel) and a raster plot of 100 randomly sampled neurons (bottom panel) for an external sinusoidal input of 100 Hz (ripple oscillation) are shown. **(D)** 1000 ms of average firing probability of 1250 Izhikevich neurons (top panel) and a raster plot (bottom panel) with approximately a 6-Hz oscillation are shown. In the top panel, spikes from an excitatory neuron and an inhibitory neuron are represented by a black dot and a gray dot, respectively. **(E)** 1000 ms of average firing probability of 1250 Izhikevich neurons (top panel) and a raster plot (bottom panel) with approximately a 40-Hz oscillation are shown.

Figures 2A–C show example spike trains and multi-unit activity of binary model neurons driven by external sinusoidal inputs of 1, 6, and 100 Hz oscillations, respectively. Izhikevich neurons also exhibited very similar activity (data not shown). It can be clearly seen that neural activity is entrained to external input. Using these two models, we investigated how the IG measures are affected by externally driven oscillations.

In the second scenario (internally induced oscillation, Figure 1B), interaction between excitatory and inhibitory neuron pools generates an oscillation. For the binary neuron model, the total input to the *i*-th excitatory neuron and the *k*-th inhibitory neuron are written as,

(12) $$\begin{array}{l} u_{i}^{E}(t)=\sum_{j}J_{ij}^{EE}x_{j}^{E}(t)+\sum_{j}J_{ij}^{EI}x_{j}^{E}(t)+H_{i}^{E},\\ u_{k}^{I}(t)=\sum_{j}J_{kj}^{II}x_{j}^{I}(t)+\sum_{j}J_{kj}^{IE}x_{j}^{I}(t)+H_{k}^{I}, \end{array}$$

where *J*^*II*^, *J*^*IE*^, and *J*^*EI*^ represent negative weights between inhibitory neurons, positive weights from excitatory neurons to inhibitory neurons, and negative weights from inhibitory neurons to excitatory neurons, respectively. The excitatory and inhibitory neurons receive constant external inputs *H*^*E*^_*i*_ and *H*^*I*^_*i*_, and maintain sustained oscillatory activity. The neuronal state was updated following the transition rate *w* in Equation 7.

For the Izhikevich model in the second scenario, a similar relationship exists for the total inputs for the *i*-th excitatory neuron and the *k*-th inhibitory neuron,

(13) $$\begin{array}{l} I_{i}^{E}(t)=\sum_{j}J_{ij}^{EE}s_{j}^{E}(t)+\sum_{j}J_{ij}^{EI}s_{j}^{E}(t)+H_{i}^{E},\\ I_{k}^{I}(t)=\sum_{j}J_{kj}^{II}s_{j}^{I}(t)+\sum_{j}J_{kj}^{IE}s_{j}^{I}(t)+H_{k}^{I}. \end{array}$$

The neuronal state was then updated following Equation 9 and the associated reset dynamics. Figures 2D,E provide examples of spike trains and multi-unit activity of Izhikevich neurons that exhibited ~6 and 40 Hz oscillations, respectively. Binary neurons also exhibited a very similar activity (data not shown). Neural activity was synchronized, but the degree of entrainment was weaker than the externally driven mechanisms. Using these two models, we investigated how the IG measures were influenced by the internally induced oscillation.

## Results

### Externally driven oscillation

We investigated the relationship between the IG measures, θ^(4, *N*)^_*i*_ and θ^(4, *N*)^_*ij*_, and the connection weights, (*J*_*ij*_ + *J*_*ji*_), using a network of 1000 binary neurons and 1000 Izhikevich neurons. We focused on the IG measures with 4th-order LLM because they have been shown to estimate connection weights (Nie and Tatsuno, 2012) and external inputs (unpublished data) within a 10% error under an equilibrium assumption. In the simulation, we kept the amplitude of external input at a value such that the overall network firing probability is relatively low (*p*_*x*_*i*__ ~ 0.1). Connection weights were set to the order of 1/*N* to prevent saturation of neuronal activity. For a binary neuron model, we used *J*_*ij*_ = 1/*N* + ε_*ij*_ where ε_*ij*_ is a random variable from a normal distribution *N*(0, 1/*N*) with a mean of 0 and the standard deviation of 1/$\sqrt{N}$. For the Izhikevich model, we restricted the simulations to a pool of only excitatory neurons to ensure that no internally induced oscillation occurred. The connection weights were assigned as *J*^*EE*^_*Ij*_ = 1/*N* + ε′_*ij*_ where ε′_*ij*_ is a random variable following uniform distribution *U*(0, 1/*N*) within the interval of [0, 1/*N*]. θ ^(4, *N*)^_*i*_ and θ^(4, *N*)^_*ij*_ were calculated by 10^6^ updates of the network. With the time resolution of 1 ms, the simulation corresponds to ~15 min of recordings. To obtain the mean and variances of the IG measures, we performed 100 independent simulations. Error bars in the figure represent the standard error of mean (SEM).

**Figure 3:**
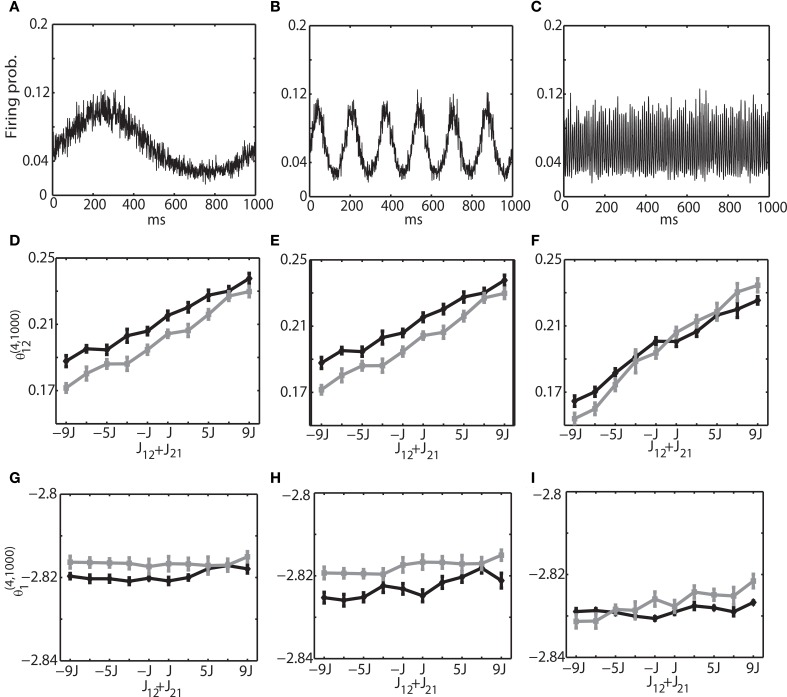
**Relationship between the IG measures and the sum of connection weights for an externally driven oscillation. (A)** Average firing probability of 1000 binary neurons with a 1-Hz oscillatory modulation is shown. **(B)** Average firing probability of 1000 binary neurons with a 6-Hz oscillatory modulation is shown. **(C)** Average firing probability of 1000 binary neurons with a 100-Hz oscillatory modulation is shown. **(D)** Relationship between the pairwise IG measure (θ^(4, 1000)^_12_) and the sum of connection weights (*J*_12_ + *J*_21_) under a 1-Hz oscillation. Black and gray lines represent the simulation results by binary neurons and Izhikevich neurons, respectively. **(E)** Relationship between the pairwise IG measure (θ ^(4, 1000)^_12_) and the sum of connection weights (*J*_12_ + *J*_21_) under a 6-Hz oscillation. **(F)** Relationship between the pairwise IG measure (θ^(4, 1000)^_12_) and the sum of connection weights (*J*_12_ + *J*_21_) under a 100-Hz oscillation. **(G)** Relationship between the single IG measure (θ^(4, 1000)^_1_) and the sum of connection weights (*J*_12_ + *J*_21_) under a 1-Hz oscillation. Black and gray lines represent the simulation results by binary neurons and Izhikevich neurons, respectively. **(H)** Relationship between the single IG measure (θ^(4, 1000)^_1_) and the sum of connection weights (*J*_12_ + *J*_21_) under a 6-Hz oscillation. **(I)** Relationship between the single IG measure (θ^(4, 1000)^_1_) and the sum of connection weights (*J*_12_ + *J*_21_) under a 100-Hz oscillation.

We investigated the oscillation frequencies that have often been observed in the brain; slow oscillation (~1 Hz), theta oscillation (6–10 Hz), and ripple oscillation (100–200 Hz). The left column of (Figures 3A,D,G) shows the results for the slow oscillation. The multi-unit activity of the binary neurons exhibits a slow oscillation of the frequency of external input (Figure 3A) and the neurons were entrained to this frequency (Figure 2A). The spiking activity of Izhikevich neurons also showed almost identical activity (data not shown). To investigate how θ^(4, *N*)^_*i*_ and θ^(4, *N*)^_*ij*_ are related to the change of connection weights, we systematically modified the sum of connection weights between two neurons (1 and 2) from −9/*N* to 9/*N*. Due to the randomness of the connectivity, focusing the neurons (1 and 2) did not affect the generality. We found that θ ^(4, *N*)^_12_ was linearly related to the sum of the connection weights, and that the values of θ^(4, *N*)^_12_ for both the binary and Izhikevich models were very close (Figure 3D, black line for a binary model and gray line for the Izhikevich model). On the other hand, θ^(4, *N*)^_1_ and θ^(4, *N*)^_2_ were independent from the change of synaptic weights (Figure 3G). These results are consistent with the previous findings under the equilibrium assumption; showing that IG measures can also provide useful insights in conditions where the network oscillates. The middle and right columns of Figure 3 show the results for theta oscillations (Figures 3B,E,H) and ripple oscillations (Figures 3C,F,I), respectively. We found that the relationship between the IG measures and connection weights was robust against a different frequency of external inputs. This confirmed that the IG measures can also provide useful information for externally driven theta and ripple oscillations.

**Figure 4:**
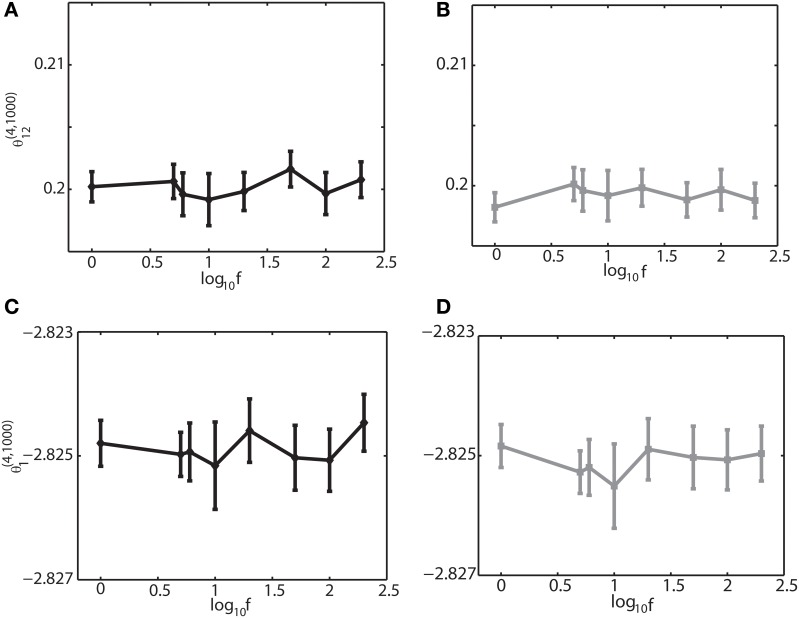
**Dependency of the IG measures for an oscillation frequency for an external drive oscillation**. The values of the single-IG measure θ^(4, 1000)^_1_ and the pairwise-IG measure θ^(4, 1000)^_12_ were calculated for different oscillatory frequencies (1, 5, 6, 10, 20, 50, 100, and 200 Hz). Parameters were set as *J*_12_ + *J*_21_ = 2*J, h*_0_ = 0.05, and φ_*i*_ = 0. **(A)** The relationship between θ^(4, 1000)^_12_ and the oscillation frequency by binary model neurons. **(B)** The relationship between θ^(4, 1000)^_12_ and the oscillation frequency by Izhikevich neurons. **(C)** The relationship between θ^(4, 1000)^_1_ and the oscillation frequency by binary model neurons. **(D)** The relationship between θ^(4, 1000)^_1_ and the oscillation frequency by Izhikevich neurons.

To further investigate if the robust property of the IG measures for slow, theta, and ripple oscillations holds true for other frequencies, we varied the frequency over 1–200 Hz, the range that can be typically observed in the brain. We set (*J*_12_ + *J*_21_) = 2*J*. Figure 4 shows that θ^(4, *N*)^_12_ and θ^(4, *N*)^_1_ did not depend on oscillation frequencies (Figures 4A,C for a binary model, and Figures 4B,D for Izhikevich model). The results confirmed that the IG measures would be useful for neural data analysis when the brain exhibits a variety of oscillations depending on cognitive demands and the sleep stages.

**Figure 5:**
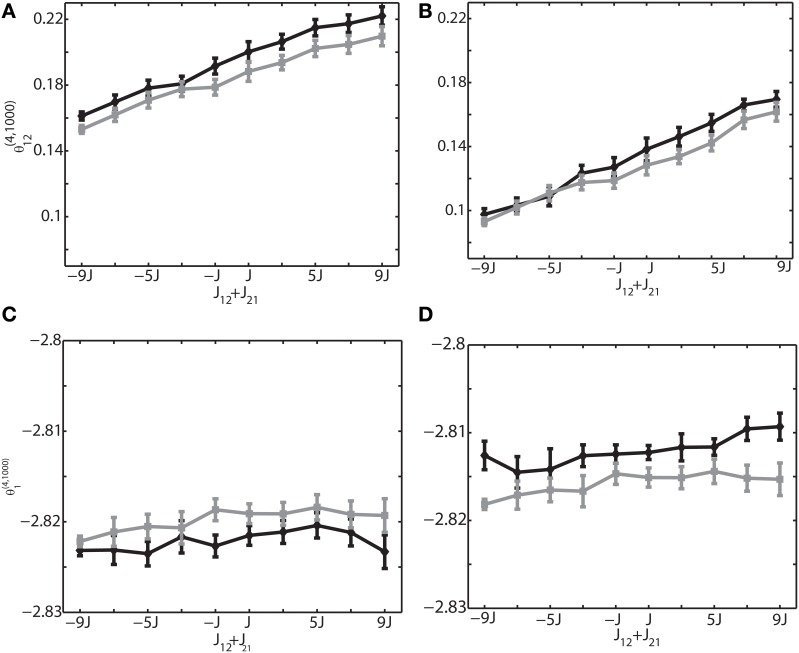
**Relationship between the IG measures and the sum of connection weights for non-zero phase differences**. An external oscillation mechanism was used, and the oscillation frequency was set to 6 Hz. **(A)** Relationship between the pairwise IG measure (θ^(4, 1000)^_12_) and the sum of connection weights (*J*_12_ + *J*_21_) for the phase difference of δφ_12_ = π/6. Black and gray curves represent the simulation by binary neurons and Izhikevich neurons, respectively. **(B)** Relationship between the pairwise IG measure θ^(4, 1000)^_12_) and the sum of connection weights, (*J*_12_ + *J*_21_) for the phase difference of δφ_12_ = π /2. **(C)** Relationship between the single-IG measure (θ^(4, 1000)^_1_) and the sum of connection weights (*J*_12_ + *J*_21_) for the phase difference of δφ_12_ = π/6. **(D)** Relationship between the single-IG measure (θ^(4, 1000)^_1_) and the sum of connection weights (*J*_12_ + *J*_21_) for the phase difference of δ φ_12_ = π /2.

The previous analyses (Figures 3, 4) were performed under the zero relative phase difference between two neurons *i* and *j*, namely δφ_*ij*_ = |φ_*i*_ − φ_*j*_| = 0. This corresponds to the synchronous neural firings that were depicted in Figures 2A–C. Neurons can, however, exhibit phase differences. For instance, sequential neural activity was observed in the natural and anesthetized brain states (Lee and Wilson, 2002; Euston et al., 2007; Luczak et al., 2007; Bermudez Contreras et al., 2013). Therefore, we calculated IG measures with phase differences. Figure 5 shows the results of the 6-Hz simulations in which the phase difference between sinusoidal inputs to the neurons 1 and 2 was set to π/6 (Figures 5A,C) and π/2 (Figures 5B,D). The rest of the neuron pairs have random phases in the range of [0, 2π]. Figures 5A,B show that θ^(4, 1000)^_12_ is linearly related to the sum of synaptic weights, suggesting that the relationship observed in zero phase difference condition also holds for the non-zero phase difference condition. Similarly, Figures 5C,D show that θ^(4, 1000)^_1_ does not depend on the connection weights, even when neurons fire with phase differences. By comparing these results with Figures 3E,H where there was no phase difference, we also found that phase difference produced the shift of the actual values of IG measures. This suggests that if the phase relationship drastically changes between the two recording epochs, the values of the IG measures cannot be directly comparable. However, if their difference is not large or if phase difference can be estimated beforehand, we could use the information for adjusting the IG values. We also confirmed that these relationships held true for slow (1 Hz) and ripple (100 Hz) frequencies (data not shown).

**Figure 6:**
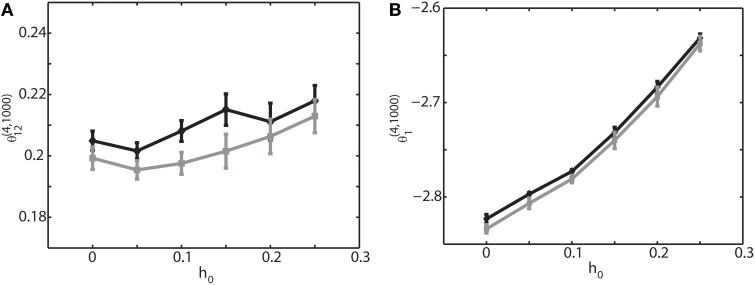
**Relationship between the IG measures and the amplitude of an external sinusoidal input for an externally driven oscillation**. Oscillation frequency was set to 6 Hz and phase difference was δφ_12_ = 0. **(A)** Relationship between the pairwise-IG measure, θ ^(4, 1000)^_12_, and the amplitude of a sinusoidal input, *h*_0_. Black and gray lines represent the simulation results by binary neurons and Izhikevich neurons, respectively. **(B)** Relationship between the single-IG measure, θ ^(4, 1000)^_1_, and the amplitude of a sinusoidal input, *h*_0_.

So far we have focused on the relationship between the IG measures and the connection weights. Another important parameter is the magnitude of sinusoidal input *h*_0_. Therefore, we have analyzed how θ^(4, 1000)^_1_ and θ^(4, 1000)^_12_ are related to *h*_0_. Figure 6 shows the result when the external sinusoidal input has a frequency of 6 Hz (theta oscillation). We found that θ^(4, 1000)^_12_ was nearly independent from the change of *h*_0_ (Figure 6A), but θ^(4, 1000)^_1_ was almost linearly related to it (Figure 6B). We also confirmed that almost identical relationship holds true for other frequencies such as slow oscillation and ripple oscillation if there is no phase difference. For non-zero phases between neurons, we observed that the IG values were shifted, like the case for the IG values and connection weights, but that the same linear and independent relationship in Figure 6 was sustained. The results under the oscillatory condition are consistent with the previous findings under the equilibrium condition; θ^(4, *N*)^_*i*_ was linearly related to the magnitude of the constant input and that θ^(4, *N*)^_*ij*_ was almost independent from it (Nie and Tatsuno, 2012). The investigation here provides further evidence that θ^(4, *N*)^_*i*_ is useful for the estimation of the magnitude of external input.

In summary, we investigated how the IG measures were influenced by an externally driven oscillation. Using a simple binary neuron model, and a more realistic Izhikevich model, we found that θ ^(4, *N*)^_*ij*_ had a linear relationship with the sum of the connection weights, and that it was almost independent from the magnitude of a sinusoidal input. In contrast, θ^(4, *N*)^_*i*_ was almost independent from the connection weights, but was linearly related to the magnitude of the sinusoidal input. These properties were not affected by the frequency of the oscillations or the relative phase differences between neurons.

### Internally induced oscillation

**Figure 7:**
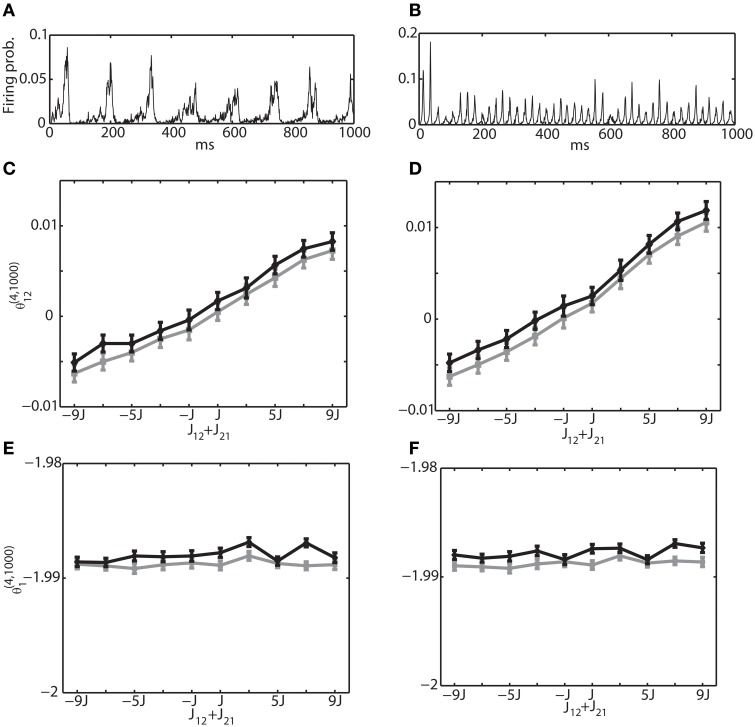
**Relationship between the IG measures and the sum of connection weights for an internally induced oscillation**. Both connections, *J*_12_ and *J*_21_, were positive for the range of (*J*_12_ + *J*_21_) ≥ 0 and they were negative for (*J*_12_ + *J*_21_) < 0. **(A)** Average firing probability of 1250 Izhikevich neurons with approximately a 6-Hz oscillatory oscillation is shown. **(B)** Average firing probability of 1250 Izhikevich neurons with approximately a 40-Hz oscillatory oscillation is shown. **(C)** Relationship between the pairwise-IG measure (θ ^(4, 1000)^_12_) and the sum of connection weights (*J*_12_ + *J*_21_) under a 6-Hz oscillation. Black and gray lines represent the simulation results by binary neurons and Izhikevich neurons, respectively. **(D)** Relationship between the pairwise-IG measure (θ^(4, 1000)^_12_) and the sum of connection weights (*J*_12_ + *J*_21_) under a 6-Hz oscillation. **(E)** Relationship between the single-IG measure (θ^(4, 1000)^_1_) and the sum of connection weights (*J*_12_ + *J*_21_) under a 6-Hz oscillation. **(F)** Relationship between the single-IG measure (θ ^(4, 1000)^_1_) and the sum of connection weights (*J*_12_ + *J*_21_) under a 40-Hz oscillation.

As another mechanism for generating an oscillatory network behavior, we also investigated the interactions between excitatory and inhibitory neuron pools. We analyzed the network structure in Figure 1B by simple binary model neurons and Izhikevich neurons. Unlike the first oscillation mechanism, where an oscillation frequency and phase differences could be explicitly controlled, it was not easy to generate an oscillation with desired parameters. However, we were able to generate two examples that were often observed in the brain. Figures 7A,B show multi-unit activity corresponding to theta frequency (~8 Hz) and gamma frequency (~40 Hz), respectively. The same examples with a raster plot were also depicted in Figures 2D,E. To avoid saturation in neural activity, we have set the connection weights to the order of 1/*N*. For a theta oscillation, we set the connection as *J*^*EE*^_*ij*_ = *J*_1_ · ϵ′_*ij*_, *J*^*IE*^_*ij*_ = 5*J*_1_ · ϵ′_*ij*_, *J*^*II*^_*ij*_ = −*J*_2_ · ϵ′_*ij*_ and *J*^*EI*^_*ij*_ = −*J*_2_ · ϵ′_*ij*_ where *J*_1_ = 1/*N*_*e*_, *J*_2_ = 1/*N_*i*_*, and ϵ′_*ij*_ is a random variable following a uniform distribution *U*(0, 1) within the interval [0,1]. For a gamma oscillation, we used *J*^*EE*^_*ij*_ = *J*_1_ · ϵ′_*ij*_, *J*^*IE*^_*ij*_ = 6*J*_1_ · ϵ′_*ij*_, *J*^*II*^_*ij*_ = −*J*_2_ · ϵ′_*ij*_ and *J^EI^_*ij*_* = −2*J*_2_ · ϵ′_*ij*_. The stronger *J*^*IE*^ was necessary to induce oscillation (Adini et al., 1997). The external constant inputs to excitatory and inhibitory neurons were set as *H*^*E*^_*i*_ = 0.05 and *H*^*I*^_*i*_ = 0.02, respectively. The simulation of 10^6^ update was performed with *N*_*e*_ = 1000 excitatory neurons and *N*_*i*_ = 250 inhibitory neurons. The mean and variance was estimated using 100 independent simulations.

To investigate the relationship between the IG measures and the sum of connection weights, without losing the generality, we modified (*J*_12_ + *J*_21_) between the neurons (1 and 2) in the range of [−9*J*, 9*J*]. Firstly, we focused on the connections within the excitatory neuron population and within the inhibitory neuron population. In other words, both connections, *J*_12_ and *J*_21_, were positive for the range of (*J*_12_ + *J*_21_) ≥ 0 and both were negative for (*J*_12_ + *J*_21_) < 0. Figures 7C,E show the relationship between θ^(4, 1250)^_12_ and θ^(4, 1250)^_1_, and the sum of connection strengths (*J*_12_ + *J*_21_) under the theta oscillation. The results clearly show that θ^(4, 1250)^_12_ is linearly related to the sum of the connection weights and that θ ^(4, 1250)^_1_ was independent from the modulation of the connection weights. Furthermore, the dependency of the IG measures on the connection weights was continuous in both positive and negative ranges. This suggests that IG measures can be applicable to both positive and negative connections. Figures 7D,F show results for gamma oscillation. Similar results were obtained for both θ^(4, 1250)^_12_ and θ^(4, 1250)^_1_.

**Figure 8:**
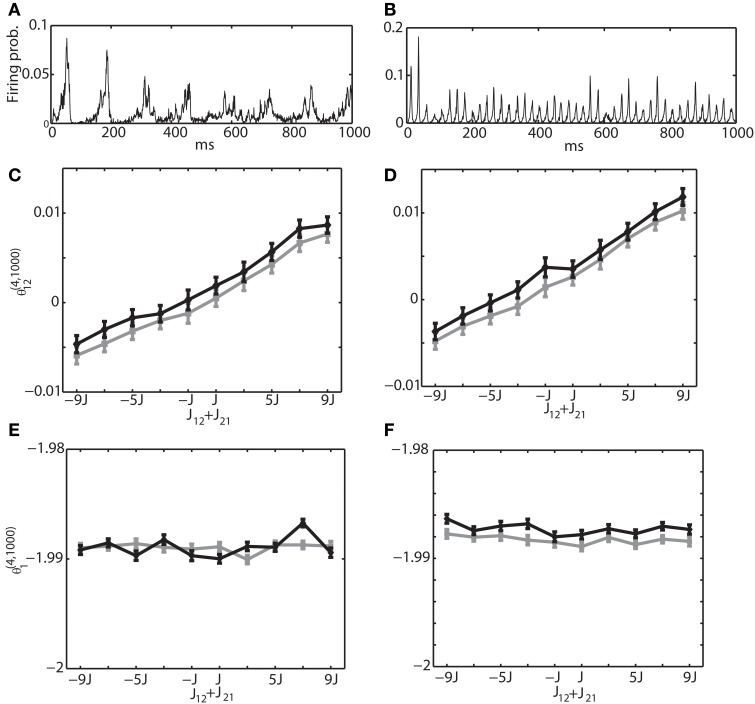
**Relationship between the IG measures and the sum of connection weights for an internally induced oscillation**. Neuron 1 was selected from the excitatory neuron pool and Neuron 2 was selected from the inhibitory neuron pool. In other words, one of the connections in *J*_12_ + *J*_21_ was positive and the other was negative. **(A)** Average firing probability of 1250 Izhikevich neurons with approximately a 6-Hz oscillatory oscillation is shown. **(B)** Average firing probability of 1250 Izhikevich neurons with approximately a 40-Hz oscillatory oscillation is shown. **(C)** Relationship between the pairwise-IG measure (θ^(4, 1000)^_12_) and the sum of connection weights (*J*_12_ + *J*_21_) under a 6-Hz oscillation. Black and gray lines represent the simulation results by binary neurons and Izhikevich neurons, respectively. **(D)** Relationship between the pairwise-IG measure (θ ^(4, 1000)^_12_) and the sum of connection weights (*J*_12_ + *J*_21_) under a 6-Hz oscillation. **(E)** Relationship between the single-IG measure (θ^(4, 1000)^_1_) and the sum of connection weights (*J*_12_ + *J*_21_) under a 6-Hz oscillation. **(F)** Relationship between the single-IG measure (θ^(4, 1000)^_1_) and the sum of connection weights (*J*_12_ + *J*_21_) under a 40-Hz oscillation.

Secondly, we investigated the interaction between excitatory and inhibitory neurons. Namely, we selected the neuron 1 from the excitatory neuron pool and the neuron 2 from the inhibitory neuron pool. The sum of connection weights was modified from −9*J* to 9*J*. Figures 8A,B are the same with Figures 7A,B, showing the multi-unit activity for theta and gamma oscillation, respectively. Figures 8C,E show the relationship between the IG measures and the sum of the connection weights under the theta oscillation. Similarly, Figures 8D,F are for gamma oscillation. The results show that the linear dependency of θ^(4, 1250)^_12_ on the sum of the connection weights holds true for an excitatory and inhibitory neuron pair. We also found that θ^(4, 1250)^_1_ had almost no relationship with the sum of connection weight. For the relationship between the IG measures and the magnitude of constant input *H*^*E*^_*i*_ and *H*^*I*^_*i*_, we confirmed that θ ^(4, 1250)^_1_ was linearly related to their magnitude, but θ^(4, 1250)^_12_ was independent from them (data not shown).

In summary, for internally generated oscillations, we demonstrated that the relationship between the IG measures and the connection weights that were found under equilibrium assumption also held true.

## Discussion

Previous studies have shown that the IG measures provided useful information about network structures (Tatsuno and Okada, 2004; Tatsuno et al., 2009; Nie and Tatsuno, 2012). Specifically, the single-IG measure θ^(4, *N*)^_*i*_ was related to the magnitude of external constant input, and the pairwise-IG measure θ^(4, *N*)^_*ij*_ was related to the sum of the connection strengths. Although these studies were conducted under the equilibrium assumption, the real neural signals exhibit various oscillations depending on cognitive demand of the task or the state of the brain. Therefore, we studied the relationship between the IG measures and the neural network parameters under oscillatory network states.

We have considered two general oscillation mechanisms; one was the oscillation driven by external input, and the other was the oscillation induced internally due to interactions between excitatory and inhibitory neuron pools. Numerical simulation was performed by the network of a simple binary neuron model and the Izhikevich neuron model. The former model was used so as to compare the results with that of previous studies, and the latter was used to investigate the relationship with more realistic model neurons.

For the external oscillation, our investigation showed that θ^(4, *N*)^_*ij*_ was linearly related to the sum of the connection strengths, and that θ^(4, *N*)^_*i*_ was independent from it over a wide range of frequency from 1 to 200 Hz. We also showed that the relationship holds true when there are phase differences between neurons. In addition, we demonstrated that θ^(4, *N*)^_*i*_ was almost linearly related to the magnitude of sinusoidal input, but that θ^(4, *N*)^_*ij*_ was almost independent from it. For the internally induced oscillation, we have also confirmed that θ^(4, *N*)^_*ij*_ was linearly related to the sum of the connection strengths, and that θ^(4, *N*)^_*i*_ was independent from it. We have also shown that the same relationship holds true for any neuron pairs (within excitatory population, within inhibitory population, and across excitatory and inhibitory populations).

In summary, this study and previous studies have demonstrated that the IG measure provides useful information for analyzing neural circuits; not only for the equilibrium condition, but also for the oscillatory condition. The single-IG measure is useful for estimating the relative strength of external inputs. In addition, the single-IG measure is better than using the change in firing rate because the firing rate can be modulated both by the change in synaptic coupling strength and the magnitude of external inputs. Studies show that the appropriately selected single-IG measure is capable of estimating the external inputs with relatively small influence from synaptic interactions. Similarly, the pairwise-IG measure can provide more direct information about the synaptic interactions between neurons than other correlation measures (Amari, 2009). It has been also shown that the pairwise-IG measure is statistically independent from the change in firing rate and that it provides pure neural interactions (Amari, 2001; Nakahara and Amari, 2002). Together with the findings in this study, the pairwise-IG measure is a very useful measure to study direct neural interactions between neurons.

This study suggests that the actual values of the IG measures depend on the mechanisms of oscillation. For an externally driven oscillation, θ^(4, *N*)^_12_ ~ 0.2 was obtained for (*J*_12_ + *J*_21_) ~ 1/*N*. For an internally induced oscillation, the same connection strength produced θ^(4, *N*)^_12_ ~ 0.002. Within the same oscillation mechanism, the selection of model neurons (binary model or Izhikevich model), or a small difference in network parameters such as phase differences also produced a difference in the actual value of the IG measures. Nonetheless, as long as the network is in one of the oscillation mechanisms, and the phase difference is kept the same, the IG measures can provide useful insights into network structures regardless of the oscillation frequencies.

In the study of memory consolidation, one of the key questions is to understand how the changes in synaptic connections are related to learning and memory formation. Evidence suggests that neural activity during slow-wave sleep plays an important role in learning (Diekelmann and Born, 2010). Specifically, there is increasing evidence supporting the hypothesis that replay of neural activity during subsequent sleep is positively correlated with memory formation (Pavlides and Winson, 1989; Wilson and McNaughton, 1994; Kudrimoti et al., 1999; Lee and Wilson, 2002; Euston et al., 2007; Girardeau et al., 2009; Peyrache et al., 2009b; Ego-Stengel and Wilson, 2010). However, the direct information about synaptic change is not available from multi-unit recordings of a freely behaving animal because spikes and local field potentials are the two main observables. In this study, we showed that θ^(4, *N*)^_*ij*_ was linearly related to the sum of the connection weights, and that θ ^(4, *N*)^_*i*_ was linearly related to the magnitude of external inputs, even under the oscillatory conditions. We have also verified these relationships not only with a simple binary model neuron, but also with a more realistic spiking model neuron. This finding would allow us to analyze neural activity during slow-wave sleep before and after the task; θ^(4, *N*)^_*ij*_ would be a good measure for the change of connection weight, and θ^(4, *N*)^_*i*_ for the magnitude of background input that would be influenced by local field potentials. By comparing the relative change of θ^(4, *N*)^_*ij*_ between slow-wave sleeps before and after the task, and the strength of memory replay/improvement of behavior performance, the IG measure may provide a way to estimate the relationship between the synaptic modification and memory formation without having direct access to information of synaptic change.

As a related approach to the IG method, the maximum entropy (MaxEnt) has attracted much attention recently (Schneidman et al., 2006; Tang et al., 2008; Tyler et al., 2012). The philosophy of the MaxEnt approach is not to assume anything other than what we know from the data. For example, if firing rate and pairwise correlation are the only information we have, the distribution with maximum entropy is given as the Boltzmann distribution,

(14) $$P^{\left(2\right)}\left\{x\right\}=\frac{1}{Z}\exp\left(\sum_{i}h_{i}^{\prime }x_{i}+\sum_{i<j}J_{ij}^{\prime }x_{i}x_{j}\right),$$

where *h*′_*i*_ is a bias term for the neuron *i*, *J*′_*ij*_ is the symmetric coupling strength between neurons *i* and *j*, and the partition function *Z* is given by,

(15) $$Z=\sum_{\left\{x\right\}}\exp\left(\sum_{i}h_{i}^{\prime }x_{i}+\sum_{i<j}J_{ij}^{\prime }x_{i}x_{j}\right).$$

We see that the MaxEnt is equivalent to the IG with the 2nd-order LLM,

(16) $$\log p_{x_{1}x_{2}*\;\cdots \;*}=\sum_{i}\theta_{i}^{\left(2,\;N\right)}x_{i}+\sum_{i<j}\theta_{ij}^{\left(2,\;N\right)}x_{i}x_{j}-\psi {\left(\theta \right)}^{\left(2,\;N\right)},$$

where the relationship between the parameters are given as,

(17) $$\theta_{i}^{\left(2,\;N\right)}=h_{i}^{\prime },\theta_{ij}^{\left(2,\;N\right)}=J^{\prime }{}_{ij},\psi {\left(\theta \right)}^{\left(\text{2},\text{ N}\right)}=\log Z.$$

As was discussed in Tatsuno and Okada (2004) and in Tatsuno et al. (2009), it is possible to relate these IG measures, θ^(2, *N*)^_*i*_ and θ^(2, *N*)^_*ij*_, to the network structure even for a network with asymmetric connections (Equation 5). However, under the influence of correlated inputs, we have also shown that the relationship in Equation 5 broke down, and that it was necessary to use the IG measures with the higher-order LLM such as the 4th-order (Equation 6) (Nie and Tatsuno, 2012). In other words, it was necessary to take into account neural activity of two additional neurons to estimate the direct neural interaction between neuron *i* and *j*. In summary, we see that the MaxEnt approach and the IG method are closely related. In addition, we also see that the MaxEnt can be considered a part of the IG method that provides a more general analysis framework for the space of the probability distributions.

In this study, we used the synchronous neural activity for estimating the direct neural interaction as the form of (*J*_*ij*_ + *J*_*ji*_). However, in the real learning processes such as sequential learning, it is possible that synaptic modification occurs differently for each direction; e.g., *J*_*ij*_ increases, while *J*_*ji*_ decreases. The proposed method is not able to estimate the directed synaptic change. As one possible remedy for this difficulty, calculation of the pairwise-IG measure using the time-lagged spiking activity between neurons was suggested (Tatsuno and Okada, 2004; Nie and Tatsuno, 2012). Another limitation of the present study is not including the effect of delay; e.g., axonal conduction delay or synaptic transmission delay. It is possible that these delays dramatically change the firing patterns as well as increase a variety of coexisting patterns (Izhikevich, 2006). Little is known about the relationship between the IG measures and direct neural interactions with conduction delay. In addition, it has not been clear how IG measures with more neuronal interactions such as triplewise-IG measures θ^(*k*, *N*)^_*ijk*_ or quadruple-IG measures θ^(*k*, *N*)^_*ijkl*_ behave under oscillatory conditions. It would be interesting to extend the current study to include more neuronal interactions.

Despite these limitations, the IG method is one of the most promising statistical tools for spike train analysis (Amari, 2001; Nakahara and Amari, 2002). Its direct relationship with the network parameters would provide useful information for the estimation of structural changes (Tatsuno and Okada, 2004; Tatsuno et al., 2009; Nie and Tatsuno, 2012). We hope that an advancement of novel analysis methods including IG will lead to a break-through finding in neuroscience.

### Conflict of interest statement

The authors declare that the research was conducted in the absence of any commercial or financial relationships that could be construed as a potential conflict of interest.
